# Editorial

**Published:** 1842-01-29

**Authors:** 


### THE MEDICAL EXAMINER.

PHILADELPHIA, JANUARY 29, 1842.

*Dr. Paine in reply to Dr. Carpenter.*—Dr. Paine, of New York, has published in the Boston Medical and Surgical Journal a rejoinder to the refutation of a charge of plagiarism, brought against Dr. Carpenter, of England, which we inserted in the fifty-second number of our last volume, (p. 829.) Dr. Paine's rejoinder occupies upwards of six pages of the Boston Journal; an amount of space that precludes our complying with his request to copy it. The gist of it is, that Dr. Paine refuses to admit Dr. Carpenter's disclaimer, backed as it is by the editor of the British and Foreign Review, on the ground, among other reasons, that the name of the actual plagiarist is withheld. We have no wish to enter into the merits of this discussion between an author and his reviewer, but we must say that we think Dr. Carpenter has satisfactorily freed himself from the stigma of literary plagiarism. We are surprised that Dr. Paine should not only not withdraw his charge, but should continue to harp upon such flimsy circumstantial evidence as mere coincidence of thought and style between Dr. Carpenter and the plagiarist, after the direct rebutting testimony which has been adduced. Irritated as Dr. Paine may feel at what he deems the injustice and misrepresentations of the British and Foreign Review, we cannot bear him out in now persisting in the gross accusation which he has brought against a gentleman of Dr. Carpenter's personal and professional reputation. As an act of simple justice, we must express the opinion,—certainly an impartial one,—that the charge falls to the ground.

